# Antileishmanial Aminopyrazoles: Studies into Mechanisms and Stability of Experimental Drug Resistance

**DOI:** 10.1128/AAC.00152-20

**Published:** 2020-08-20

**Authors:** M. Van den Kerkhof, D. Mabille, S. Hendrickx, P. Leprohon, C. E. Mowbray, S. Braillard, M. Ouellette, L. Maes, G. Caljon

**Affiliations:** a Laboratory of Microbiology, Parasitology and Hygiene (LMPH), University of Antwerp, Antwerp, Belgium; b Drugs for Neglected Diseases initiative (DNDi), Geneva, Switzerland; c Centre de Recherche en Infectiologie du Centre de Recherche du Centre Hospitalier Universitaire de Québec, Université Laval, Québec, Québec, Canada

**Keywords:** *Leishmania*, ABC transporters, aminopyrazoles, resistance

## Abstract

Current antileishmanial treatment is hampered by limitations, such as drug toxicity and the risk of treatment failure, which may be related to parasitic drug resistance. Given the urgent need for novel drugs, the Drugs for Neglected Diseases *initiative* (DND*i*) has undertaken a drug discovery program, which has resulted in the identification of aminopyrazoles, a highly promising antileishmanial chemical series. Multiple experiments have been performed to anticipate the propensity for resistance development. Resistance selection was performed by successive exposure of Leishmania infantum promastigotes (*in vitro*) and intracellular amastigotes (both *in vitro* and in golden Syrian hamsters). The stability of the resistant phenotypes was assessed after passage in mice and Lutzomyia longipalpis sandflies. Whole-genome sequencing (WGS) was performed to identify mutated genes, copy number variations (CNVs), and somy changes. The potential role of efflux pumps (the MDR and MRP efflux pumps) in the development of resistance was assessed by coincubation of aminopyrazoles with specific efflux pump inhibitors (verapamil, cyclosporine, and probenecid). Repeated drug exposure of amastigotes did not result in the emergence of drug resistance either *in vitro* or *in vivo*. Selection at the promastigote stage, however, was able to select for parasites with reduced susceptibility (resistance index, 5.8 to 24.5). This phenotype proved to be unstable after *in vivo* passage in mice and sandflies, suggesting that nonfixed alterations are responsible for the elevated resistance. In line with this, single nucleotide polymorphisms and indels identified by whole-genome sequencing could not be directly linked to the decreased drug susceptibility. Copy number variations were absent, whereas somy changes were detected, which may have accounted for the transient acquisition of resistance. Finally, aminopyrazole activity was not influenced by the MDR and MRP efflux pump inhibitors tested. The selection performed does not suggest the rapid development of resistance against aminopyrazoles in the field. Karyotype changes may confer elevated levels of resistance, but these do not seem to be stable in the vertebrate and invertebrate hosts. MDR/MRP efflux pumps are not likely to significantly impact the activity of the aminopyrazole leads.

## Abstract (precis)

Current antileishmanial treatment is hampered by limitations, such as drug toxicity and the risk of treatment failure, which may be related to parasitic drug resistance. Given the urgent need for novel drugs, the Drugs for Neglected Diseases *initiative* (DND*i*) has undertaken a drug discovery program, which has resulted in the identification of aminopyrazoles, a highly promising antileishmanial chemical series. Multiple experiments have been performed to anticipate the propensity for resistance development.

## INTRODUCTION

Visceral leishmaniasis (VL) is a disease generally caused by the protist parasites Leishmania infantum and Leishmania donovani, transmitted by the bite of infected sandflies (1, 2). The *Leishmania* parasite has a dimorphic life cycle, with a motile promastigote stage in the vector and a nonmotile amastigote stage in host myeloid cells. Infection can disseminate to the liver, spleen, and bone marrow, causing severe symptoms, such as prolonged fever, hepatosplenomegaly, pancytopenia, progressive anemia, and weight loss. VL can, therefore, be fatal without adequate treatment (3). Currently available therapies are limited, as they either lack sufficient effectiveness, show important levels of toxicity, or have to be administered parenterally. Drug resistance might also be a concern and could affect the efficacy of current and future new drugs in the field. Antimonials (Sb) have been the therapeutic option of choice for several decades but are no longer effective in the Indian subcontinent due to widespread resistance (4). Miltefosine (MIL), currently the only available oral drug, is confronted with increasing treatment failure rates (5–9). A few clinical isolates with reduced susceptibility to amphotericin B (AmB) have also been found (10). The emergence of drug resistance can often be ascribed to the ability of the parasite to overcome the cytotoxic effects of drugs either by causing mutations that alter the drug target or the uptake system or by overexpressing efflux pumps (11). A well-known efflux pump family is the ATP-dependent binding cassette (ABC) transporter superfamily (ABC transporters), which is located in extra- or intracellular membranes and which can extrude a wide variety of substrates. The upregulation of these transporters has already been linked to drug resistance in *Leishmania* (12, 13). More particularly, the upregulation of ABCC3 (MRPA or PGPA) and ABCC7 (PRP1) (14–16) and ABCB1 (MDR or PGP) (17) contributes significantly to various degrees of Sb resistance. Also, the reported AmB resistance has been linked to the overexpression of ABCB1 (10). Other efflux pumps of the same superfamily, such as ABCG4 and ABCG6, have also been identified as playing a role in drug resistance (18, 19). Additionally, host cell ABC transporters can codetermine the susceptibility of intracellular *Leishmania* stages (20), as these may regulate intracellular drug availability. For example, reduced drug exposure in Sb-resistant parasites was linked to the upregulation of the ABCC1 pump in macrophages (21). One approach to studying the involvement of ABC transporters in *Leishmania* drug resistance is the use of specific efflux pump inhibitors to reverse resistance (22–24).

To summarize, there is a crucial need for the development of novel drugs that are affordable, easy to use, and safe and that preferably have an alternative mode of action to the currently available antileishmanials. The latter is important for the possible implementation of combination therapies, which could result in improved efficacy, reduced side effects, a lower risk of resistance emergence, and shorter treatment durations (2, 25–27). Several new drug classes that have good efficacy against *Leishmania* have been discovered, including nitroimidazoles, oxaboroles, proteasome inhibitors, cdc2-related kinase 12 (CRK-12) inhibitors, and aminopyrazoles (APs) (25). Simultaneously, efforts to determine their properties and mode of action have been or are being undertaken (25, 28–30). However, aside from the pharmacodynamics *in vitro* and *in vivo* (28, 31), not much is known about the antileishmanial action of the AP class. One study has identified mitogen-activated protein kinases (MAPK) and cdc2-related kinases (CRK) to be potential target proteins of a diaminopyrimidine in Trypanosoma brucei and Leishmania major (32). Other distant analogues have been used in oncology and shown to inhibit human cyclin-dependent kinases (CDK) (33–35) and posttranslational modification processes (36). In Plasmodium falciparum, mutations in a P-type ATPase were shown to confer resistance to GNF-Pf4492, a pyrazole urea (37). All these studies have been performed with distant analogues and may provide only some indication for the antileishmanial effects. Additionally, it is essential to explore the possibility of the rapid development of resistance against novel compounds, as this may severely impact a drug’s clinical life span once it is widely used in the field. The number of alternatives in the drug discovery pipeline is rather scarce, and this kind of information is important to safeguard novel drugs from the emergence of resistance, to guide dosing regimens, and to predict the importance of incorporation in a combination therapy.

The aim of this laboratory study was therefore (i) to evaluate the propensity of VL species to develop resistance to the new AP lead series, (ii) to characterize the resistant strains obtained, and (iii) to check the involvement of the major efflux pumps MDR and MRP in both the parasite and the macrophage host cell.

## RESULTS

### *In vitro* and *in vivo* resistance selection on the amastigote stage.

Selection for resistance to the AP series was performed to evaluate the possible propensity toward resistance to these compounds in the field and to allow mode-of-action/resistance studies. Both *in vitro* and *in vivo* intracellular resistance selection procedures have been shown to be successful in previous studies (38, 39). *In vitro* resistance selection by five successive cycles of exposure of intracellular L. infantum amastigotes (using laboratory strain MHOM/MA/67/ITMAP263 [ITMAP263] and field strain MHOM/FR/09/LEM4038 [LEM4038]) to the AP lead compound DNDI-1044 did not result in significant differences in 50% inhibitory concentration (IC_50_) values between the wild-type (WT) parent and the selected parasites (Table 1). Similarly, resistance selection of the lab strain (ITMAP263) in golden Syrian hamsters failed to induce a shift in the IC_50_ for DNDI-1044 when the IC_50_s for the wild-type parent (0.46 ± 0.09 μM) and the *ex vivo* amastigotes collected after five successive selection cycles (0.42 ± 0.07 μM) were compared.

**TABLE 1:** *In vitro* susceptibility of intracellular amastigotes to DNDI-1044 after each *in vitro* selection cycle^*a*^

Cycle	Mean IC_50_ (μM) ± SEM	
	ITMAP263	LEM4038
Wild type	0.19 ± 0.08	0.35 ± 0.07
Cycle 1	0.21 ± 0.02	0.43 ± 0.08
Cycle 2	0.60 ± 0.07	0.27 ± 0.04
Cycle 3	0.45 ± 0.09	0.36 ± 0.11
Cycle 4	0.53 ± 0.15	0.56 ± 0.17
Cycle 5	0.24 ± 0.08	0.86 ± 0.30

a The results are based on two independent replicates run in duplicate.

### *In vitro* resistance selection on the promastigote stage.

**FIG 1:**
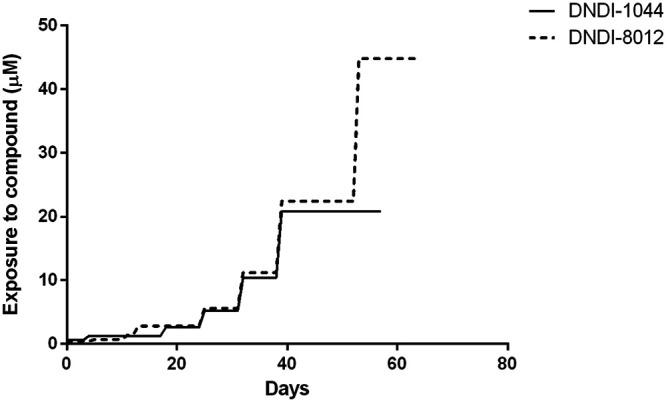
Overview of the stepwise generation of extracellular promastigote lines resistant to two APs.

As resistance selection in the relevant host amastigote stage failed, an attempt was made in the extracellular insect promastigote stage using the L. infantum MHOM/FR/96/LEM3323 (LEM3323) clinical isolate. Selection for resistance to the two selected APs was successful within approximately 60 days. Drug exposure could be increased in a stepwise fashion to a maximum of 20 μM and 45 μM for DNDI-1044 and DNDI-8012, respectively (Fig. 1). Higher drug concentrations led to parasite death. The stepwise selection showed a gradual increase in the IC_50_ upon each successive passage, with a significant increase being seen after 4 passages in the extracellular assay (see Fig. S1 in the supplemental material). Selection for resistance to both DNDI-1044 and DNDI-8012 was therefore successful, with the resistance indexes (RI) being 24.5 and 5.8, respectively. Cross-resistance was observed for both resistant lines (Table 2). An intracellular susceptibility assay was performed to assess resistance at the amastigote stage. Only the DNDI-1044-selected resistant line (LEM3323Cl_4_/1044) retained its resistant and cross-resistant phenotype at the amastigote stage (Table 2; Fig. S2). These resistant strains selected in the promastigote stage were used for further characterization.

**TABLE 2:** Resistance index for the AP-selected lines after the highest exposure^*a*^

Compound	Extracellular promastigote assay						Intracellular amastigote assay					
	Wild type vs LEM3323Cl_4_/1044			Wild type vs LEM3323Cl_4_/8012			Wild type vs LEM3323Cl_4_/1044			Wild type vs LEM3323Cl_4_/8012		
	Mean IC_50_ (μM) ± SEM		RI	Mean IC_50_ (μM) ± SEM		RI	Mean IC_50_ (μM) ± SEM		RI	Mean IC_50_ (μM) ± SEM		RI
	Wild type	LEM3323Cl_4_/1044		Wild type	LEM3323Cl_4_/8012		Wild type	LEM3323Cl_4_/1044		Wild type	LEM3323Cl_4_/8012	
DNDI-1044	0.20 ± 0.04	4.90 ± 0.55	24.5	0.34 ± 0.11	2.78 ± 0.55	8.2	0.44 ± 0.12	2.01 ± 0.46	4.6	0.29 ± 0.01	0.37 ± 0.01	1.3
DNDI-8012	0.52 ± 0.02	7.55 ± 1.30	14.5	0.62 ± 0.12	3.62 ± 0.31	5.8	0.54 ± 0.10	2.20 ± 0.35	4.1	0.22 ± 0.00	0.19 ± 0.01	0.9

a The results are based on two independent replicates run in duplicate. The intracellular assay with LEM3323Cl_4_/8012 was performed only once in duplicate, as no differences were observed. RI, resistance index.

### Generation of clones.

**FIG 2:**
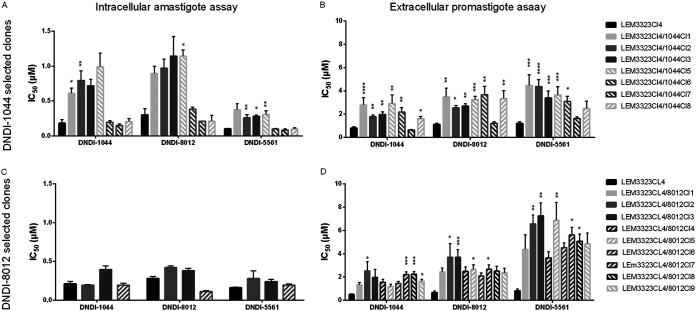
Comparison of *in vitro* intracellular amastigote (A and C) and extracellular promastigote (B and D) susceptibility to the selected AP series between the wild-type parent (LEM3323Cl_4_) and the successfully cultured adapted clones of the DNDI-1044-selected (A and B) and DNDI-8012-selected (C and D) resistant lines. Results are expressed as the mean IC_50_ ± SEM and are based on two independent replicates run in duplicate (*, *P* < 0.05; **, *P* < 0.01; ***, *P* < 0.001; ****, *P* < 0.0001). For DNDI-8012-generated clones, only a few clones were selected for amastigote susceptibility assessment.

The microdrop technique was used to obtain individual clones of the two resistant strains, to reduce variability in further experiments. A total of nine clones (LEM3323Cl_4_/8012Cl_1_ [8012Cl_1_] to LEM3323Cl_4_/8012Cl_9_ [8012Cl_9_]) were generated from the DNDI-8012-selected line (LEM3323Cl_4_/8012), and seven more clones (LEM3323Cl_4_/1044Cl_1_ [1044Cl_1_] to LEM3323Cl_4_/1044Cl_7_ [1044Cl_7_]) were obtained from the DNDI-1044-selected resistant L. infantum line (LEM3323Cl_4_/1044). Despite the slight decrease in RI that could be observed after the cloning procedure, the resistance profile in the promastigote parasite stage was maintained for both selected lines (Fig. 2; Table 3). One line, namely, LEM3323Cl_4_/1044Cl_7_, did not show a resistant phenotype. Cross-resistance was observed for all tested APs, which also included the new lead DNDI-5561. No resistant phenotype was observed for the intracellular amastigotes of the LEM3323Cl_4_/8012 clones, which was expected, given the susceptible characteristic of the polyclonal parent line. The polyclonal LEM3323Cl_4_/1044-selected line, on the contrary, did show a resistant phenotype with an RI of ±4 for intracellular amastigotes. However, the full resistant phenotype could not be maintained with some of the clones displaying an intermediate resistant phenotype (clones LEM3323Cl_4_/1044Cl_1_, LEM3323Cl_4_/1044Cl_2_, LEM3323Cl_4_/1044Cl_3_, and LEM3323Cl_4_/1044Cl_5_). The LEM3323Cl_4_/1044Cl_1_ line was the first clone to be generated and was used in further experiments. In the resistant clones, cross-resistance was observed for all tested APs (Fig. 2; Table 4).

**TABLE 3:** Resistance index for the AP-selected lines in the extracellular promastigote assay^*a*^

Compound	Wild type vs LEM3323Cl_4_/1044Cl_1_			Wild type vs LEM3323Cl_4_/8012Cl_2_		
	Mean IC_50_ (μM) ± SEM		RI	Mean IC_50_ (μM) ± SEM		RI
	Wild type	LEM3323Cl_4_/1044Cl_1_		Wild type	LEM3323Cl_4_/8012Cl_2_	
DNDI-1044	0.50 ± 0.06	2.79 ± 0.61	5.6	0.50 ± 0.06	2.52 ± 0.81	5.0
DNDI-8012	0.67 ± 0.12	3.48 ± 0.75	5.2	0.67 ± 0.12	3.70 ± 1.17	5.5
DNDI-5561	0.82 ± 0.16	4.45 ± 0.93	5.4	0.82 ± 0.16	6.57 ± 0.76	8.0

a Results are based on two independent replicates run in duplicate. RI, resistance index.

**TABLE 4:** Resistance index for the AP-selected lines in the intracellular amastigote assay^*a*^

Compound	Wild type vs LEM3323Cl_4_/1044Cl_1_			Wild type vs LEM3323Cl_4_/8012Cl_2_		
	Mean IC_50_ (μM) ± SEM		RI	Mean IC_50_ (μM) ± SEM		RI
	Wild type	LEM3323Cl_4_/1044Cl_1_		Wild type	LEM3323Cl_4_/8012Cl_2_	
DNDI-1044	0.18 ± 0.05	0.61 ± 0.08	3.4	0.21 ± 0.03	0.20 ± 0.01	1.0
DNDI-8012	0.30 ± 0.09	0.90 ± 0.10	3.0	0.28 ± 0.02	0.42 ± 0.02	1.5
DNDI-5561	0.10 ± 0.003	0.37 ± 0.09	3.6	0.16 ± 0.01	0.28 ± 0.10	1.8

a Results are based on two independent replicates run in duplicate. RI, resistance index.

### Intracellular multiplication of the resistant lines.

**FIG 3:**
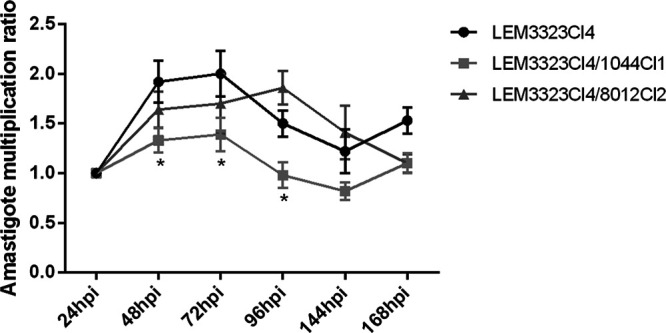
Comparison of the *in vitro* amastigote multiplication ratio between the wild-type parent (LEM3323Cl_4_) and the selected resistant lines. Results are expressed as the mean amastigote multiplication ratio ± SEM and are based on one experiment run in sextuplet (*, *P* < 0.05).

Given that drug-resistant traits could affect parasite infectivity, the amastigote multiplication ratio was calculated over a period of 7 days to evaluate the intracellular expansion of the resistant lines compared to that of the wild type (Fig. 3). The number of amastigotes in at least 50 macrophages was counted, and the ratio was used to determine the infection ratio (IR) for each time point. The amastigote multiplication ratio was determined by normalization using the initial infection index at 24 h postinfection (hpi). The IR and the percentage of infected macrophages at 24 hpi can be found in Table S2. A slight decrease in amastigote multiplication could be noted for the DNDI-1044-selected resistant clone, whereas the DND-8012-selected strain did not show significant differences from the WT.

### Stability of the resistant phenotype.

To assess the stability of the resistant phenotype, the generated resistant clones LEM3323Cl_4_/1044Cl_1_ and LEM3323Cl_4_/8012Cl_2_ were used for infection in both mouse and sandfly. The susceptibility was assessed before and after *in vivo* passage and compared to that of the wild-type line passaged under the same conditions. This experiment could give some insights into the possibility that the resistant phenotype would be sustained in the field. The fully resistant phenotype of promastigotes, which was clearly present prior to the *in vivo* passage, was lost after infection in mouse and sandfly. This loss was complete for LEM3323Cl_4_/1044Cl_1_, but an intermediate resistant phenotype could still be observed for LEM3323Cl_4_/8012Cl_2_ (Table 5).

**TABLE 5:** Comparison of *in vitro* extracellular promastigote susceptibility to the selected AP series before and after *in vivo* passage (mouse and sandfly) between the wild type and the generated clones

Time relative to passage	Compound	Mean IC_50_ (μM) ± SEM^*a*^		
		Wild type	LEM3323Cl_4_/1044Cl_1_	LEM3323Cl_4_/8012Cl_2_
Original	DNDI-1044	0.46 ± 0.05	3.37 ± 0.54 (7.3)****	2.63 ± 0.25 (5.7)***
	DNDI-8012	0.66 ± 0.09	4.12 ± 0.89 (6.2)****	3.81 ± 0.43 (5.7)****
	DNDI-5561	0.80 ± 0.08	9.28 ± 1.01 (11)	7.68 ± 1.09 (9.6)*
After mouse passage	DNDI-1044	0.61 ± 0.09	0.64 ± 0.10 (1.0)	1.86 ± 0.15 (3.0)
	DNDI-8012	0.73 ± 0.15	1.01 ± 0.06 (1.4)	2.68 ± 0.29 (3.7)
	DNDI-5561	0.68 ± 0.05	0.95 ± 0.16 (1.4)	2.18 ± 0.54 (3.2)
After sandfly passage	DNDI-1044	0.51 ± 0.07	0.55 ± 0.10 (1.1)	1.36 ± 0.27 (2.6)
	DNDI-8012	0.78 ± 0.12	0.85 ± 0.13 (1.1)	1.76 ± 0.17 (2.2)*
	DNDI-5561	0.74 ± 0.08	0.76 ± 0.07 (1.0)	1.20 ± 0.39 (1.6)

a Results are based on three independent replicates run in duplicate. The resistance index (RI) is given in parentheses and was always calculated with respect to the corresponding wild type. *, *P* < 0.05; ***, *P* < 0.001; ****, *P* < 0.0001.

### Comparative WGS.

Comparative whole-genome sequencing (WGS) was performed to compare both resistant strains to the WT line to unravel possible mechanisms which caused the occurrence of the resistant phenotype. A coverage of at least 121 times was obtained for all the sequenced reads. A first analysis was performed to determine the presence of single nucleotide polymorphisms (SNPs) and indels. A total of 176 and 104 nonsynonymous SNPs in coding genes was identified in LEM3323Cl_4_/1044Cl_1_ and LEM3323Cl_4_/8012Cl_2_, respectively, compared to the sequence of the reference genome, and these were absent in the wild-type line. Ten and 12 genes, respectively, were found to be mutated in a homozygous manner. Twenty genes were commonly mutated in both resistant lines, and five of these were homogeneously mutated (Table 6). Surprisingly, the same mutations were detected in all listed genes for both resistant clones, suggesting that these mutations are most likely to occur as natural polymorphisms in the parental strain. As the resistant phenotype was found to be unstable after mouse and sandfly passage, additional analysis was conducted to evaluate the possible presence of copy number variations (CNVs) in both resistant lines (see the supplemental material). No significant deletions or amplifications were noted; however, the resistant lines demonstrated aneuploidy compared to the ploidy of the parent line (Table 7).

**TABLE 6:** Overview of common mutated genes in both LEM332Cl_4_/1044Cl_1_ and LEM3323Cl_4_/8012Cl_2_^*a*^

LEM3323Cl_4_ strain	Gene identifier	Position of mutation	Ref/mut nucleotide	Allele frequency	Ref/mut AA	Gene function
1044Cl_1_	LinJ.03.0410	156	GA/G	Hom.	NA	60S acidic ribosomal protein P2, putative
8012Cl_2_		156	GA/G	Hom.	NA	
1044Cl_1_	LinJ.10.0390	172	G/A	Hom.	E/K	Folate biopterin transporter, putative
8012Cl_2_		172	G/A	Hom.	E/K	
1044Cl_1_	LinJ.12.0070	1277	G/C	Het.	R/P	Hypothetical protein, unknown function
8012Cl_2_		1277	G/C	Het.	R/P	
1044Cl_1_	LinJ.14.1180	2511	G/C	Het.	E/D	Kinesin K39, putative
8012Cl_2_		1885	T/G	Het.	S/A	
8012Cl_2_		2511	G/C	Het.	E/D	
1044Cl_1_	LinJ.14.1190	3770	A/C	Het.	E/A	Kinesin K39, putative
8012Cl_2_		3770	A/C	Het.	E/A	
8012Cl_2_		5699	G/A	Hom.	S/N	
1044Cl_1_	LinJ.15.0290	2429	T/G	Het.	L/R	Hypothetical protein, conserved
8012Cl_2_		561	C/CGTCTCGGAAGCGGAGTCGCTCTCAGCCGCG	Het.	NA	
1044Cl_1_	LinJ.18.1290	283	T/G	Het.	S/A	Hypothetical protein, conserved
8012Cl_2_		283	T/G	Het.	S/A	
1044Cl_1_	LinJ.19.1690	957	AGCGCCCCAGCCGAGCGAGGCGGCGCCGGTGTCTGCAGTGGAGGCTCTGCCTCCGACGCCTGCCGAGTGCGCATCTGAGGCG/A	Het.	NA	Hypothetical protein
8012Cl_2_		957	AGCGCCCCAGCCGAGCGAGGCGGCGCCGGTGTCTGCAGTGGAGGCTCTGCCTCCGACGCCTGCCGAGTGCGCATCTGAGGCG/A	Het.	NA	
1044Cl_1_	LinJ.27.2060	294	T/TG	Het.	NA	Hypothetical protein, unknown function
8012Cl_2_		294	T/TG	Het.	NA	
1044Cl_1_	LinJ.29.0270	546	T/G	Het.	E/D	Hypothetical protein, conserved
8012Cl_2_		546	T/G	Het.	E/D	
8012Cl_2_		544	C/G	Het.	E/Q	
1044Cl_1_	LinJ.29.2240	1267	G/A	Hom.	L/F	Hypothetical protein, conserved
8012Cl_2_		1267	G/A	Hom.	L/F	
1044Cl_1_	LinJ.31.1290	3139	G/C	Het.	R/G	P-glycoprotein e ABCC4
8012Cl_2_		3350	A/C	Het.	L/R	
8012Cl_2_		3139	G/C	Het.	R/G	
1044Cl_1_	LinJ.32.1310	827	G/C	Het.	S/T	Ubiquitin hydrolase, putative; cysteine peptidase Clan CA family C19, putative
8012Cl_2_		827	G/C	Het.	S/T	
1044Cl_1_	LinJ.33.3230	5873	A/G	Het.	Y/C	Hypothetical protein, conserved
8012Cl_2_		5873	A/G	Het.	Y/C	
1044Cl_1_	LinJ.34.0710	2417	T/C	Het.	K/R	Flagellar attachment zone protein, putative
8012Cl_2_		2417	T/C	Het.	K/R	
1044Cl_1_	LinJ.34.2650	544	T/G	Het.	S/A	Amastin-like surface protein, putative
8012Cl_2_		544	T/G	Het.	S/A	
1044Cl_1_	LinJ.35.0490	11527	G/A	Het.	A/T	Proteophosphoglycan ppg4
8012Cl_2_		11527	G/A	Het.	A/T	
1044Cl_1_	LinJ.35.0510	721	G/A	Hom.	A/T	Proteophosphoglycan ppg4
8012Cl_2_		721	G/A	Het.	A/T	
8012Cl_2_		733	C/G	Het.	L/V	
1044Cl_1_	LinJ.35.0520	6859	G/A	Het.	G/S	Proteophosphoglycan ppg4
8012Cl_2_		6859	G/A	Het.	G/S	
1044Cl_1_	LinJ.35.0540	685	T/C	Het.	C/R	Proteophosphoglycan 5
8012Cl_2_		685	T/C	Het.	C/R	

a Ref/mut, reference/mutated; Hom., homozygous; Het., heterozygous; NA, not applicable due to frameshift; AA, amino acid.

**TABLE 7:**
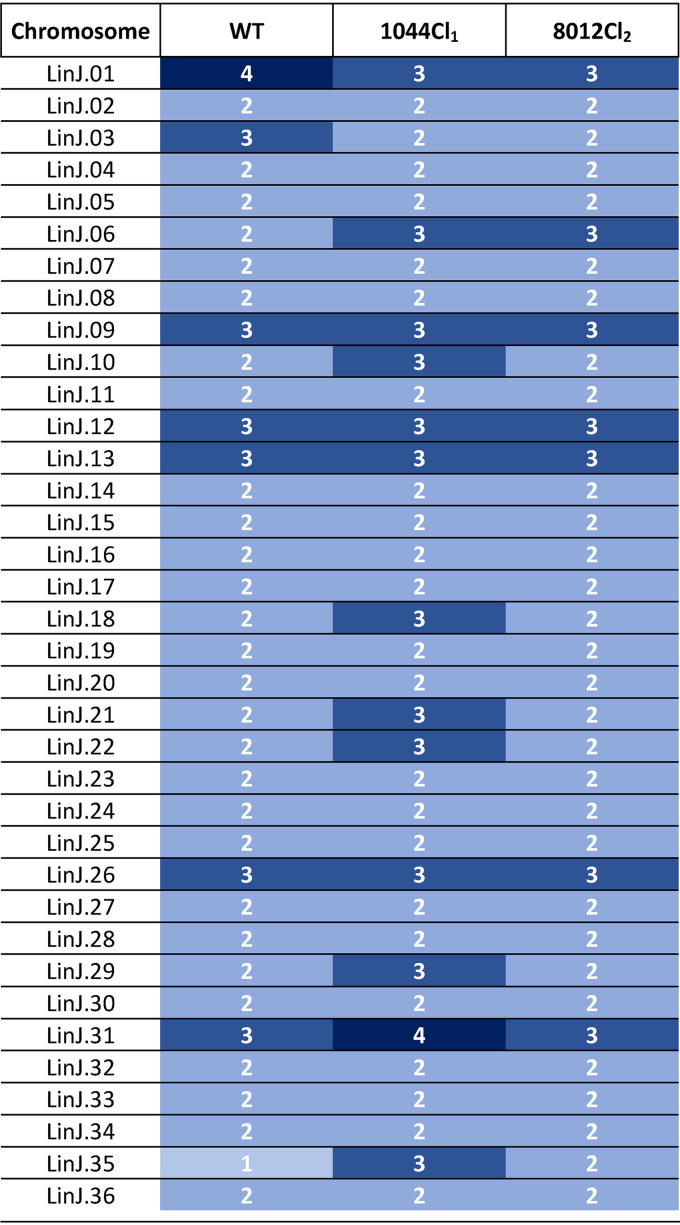
Overview of the ploidy of the wild-type line and both resistant clones^*a*^

a Chromosomal copy numbers are indicated with numbers and shades of blue ranging from light blue (mono- and disomic) to dark blue (tri- and tetrasomic). WT, wild type.

### Involvement of efflux pump inhibitors.

**FIG 4:**
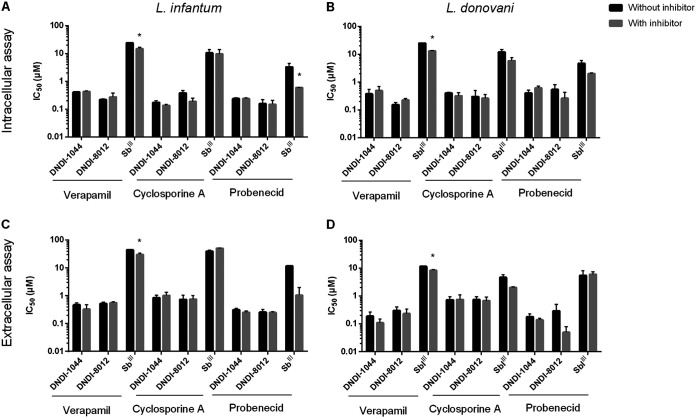
Effect of coincubation of verapamil, cyclosporine, and probenecid with the aminopyrazoles for L. infantum (A and C) and L. donovani (B and D) in the intracellular amastigote (A and B) and extracellular promastigote (C and D) susceptibility assays. Results are based on two independent repeats run in duplicate and are expressed as the mean IC_50_ ± standard mean of error (SEM) (*, *P* < 0.05).

MDR and MRP are well-known efflux pumps, which are often implicated in resistance. Their involvement was therefore determined using efflux pump inhibitors. The ABC transport inhibitors were first evaluated for their cytotoxicity on MRC5 cells and their intrinsic inhibitory effect on intracellular amastigotes and extracellular promastigotes (Table S3). Test concentrations used for the coadministration of verapamil (8 μM), cyclosporine (1.5 μM for L. infantum and 2 μM for L. donovani), and probenecid (700 μM) were selected for the susceptibility assays with the lead compounds. Under these conditions, inhibition by the ABC transporter inhibitors remained under 15%, 10%, and 20% respectively. As part of the assay validation, verapamil and probenecid were confirmed to increase the susceptibility to the trivalent form of Sb (Sb^III^), but no impact was observed with cyclosporine (Fig. 4). When the aminopyrazoles were coincubated with the ABC inhibitors, no alterations in the IC_50_ for intracellular and extracellular parasites were noted (Fig. 4). The involvement of MRP-based efflux at the host cell level was also evaluated using the fluorescent substrate 5(6)-carboxy-2′-7′-dichlorofluorescein (CDCF). CDCF efflux was inhibited by the MRP inhibitor probenecid and the MRP substrate Sb^III^, particularly in noninfected macrophages (Fig. S3). In contrast, the aminopyrazoles did not impact the efflux of CDCF by uninfected and L. infantum-infected macrophages, suggesting the absence of substrate-level competition.

## DISCUSSION

In particular areas where VL is endemic and where the use of Sb and MIL has been implemented, the effectiveness of these drugs has been gradually decreasing, as reflected by posttreatment relapses (40). It is therefore a prerequisite for any new drug under development to have a novel mode of action and a low propensity for rapid resistance development. The aminopyrazoles constitute a series, newly developed by DND*i*, which has already been shown not to have cross-resistance with the current antileishmanial drugs (28). The present study attempted to experimentally select and characterize AP-resistant strains and to evaluate the putative involvement of specific efflux pumps.

To generate experimental resistance in *Leishmania*, two approaches may be adopted: either selection of resistance on the extracellular insect stage, the promastigote, or selection on the intracellular host stage, the amastigote. The first is more regularly performed, as it is easier, cheaper, and quicker (30, 41, 42), but a major disadvantage is that it does not mimic the real interaction between the parasite and the drug in field situations. However, obtaining resistant promastigotes may still be very useful to deconvolute drug mechanisms that are shared between parasite life cycle stages. An alternative approach relies on resistance selection on the more relevant intracellular amastigote stage (38, 39). Using such a strategy, amastigote-selected drug-resistant parasite lines have already been successfully obtained for MIL and paromomycin (38, 39, 43). Selection for resistance to MIL proved to be very tedious and was successful *in vitro* for only one particular clinical isolate (43), which may reflect the situation in the field, where MIL-resistant parasite phenotypes are very scarce (44). In contrast, paromomycin resistance selection was alarmingly fast both *in vitro* and *in vivo* in multiple *Leishmania* strains, which advocates for its vigilant use, especially in monotherapy (38, 39). Exposure of amastigotes to APs *in vitro* and *in vivo* for 5 successive selection cycles failed to demonstrate changes in drug susceptibility, suggesting a low likelihood of rapid induction of resistance in the field. On the contrary, selection on promastigotes was successful after several rounds of exposure to increasing drug concentrations, allowing further phenotypic and genotypic characterization. Previous studies have demonstrated that the infectivity of drug-resistant parasites can be affected (45–49), and this was found to be decreased in one of the AP-resistant strains.

*Leishmania* parasites have developed several adaptation mechanisms to overcome elimination by drug exposure. Genetic changes, such as the introduction of SNPs and indels in drug targets or transporters, can be at the basis of a stable resistance (50–54). The AP-selected strains were found not to have a stable resistant phenotype after *in vivo* passage, suggesting that nonfixed alterations are responsible for the elevated resistance observed. Gene modulation through DNA CNVs is another way for the parasite to overcome the cytotoxic effects of a compound by specifically amplifying or deleting certain loci coding for genes involved in the drug’s mode of action or mode of resistance (55–60). In this study, the repeated exposure to APs did not give rise to CNVs; however, changes in the ploidy could be noted. Aneuploidy has been shown to be very common in *Leishmania* and has been linked to the development of resistance in a number of cases (58, 60–65). Ploidy has also been demonstrated to be rather plastic and adaptive to as little as just one *in vivo* passage (66). This could explain the resistant phenotype observed in the AP-selected parasites and the subsequent loss of resistance after a single mouse and sandfly passage. Additional generation of a larger panel of resistant parasite lines in combination with WGS could help establish the relevance of ploidy and further pinpoint the chromosomes that are most likely involved in the transient AP resistance.

Because the MDR and MRP pumps are known to confer elevated Sb^III^ and AmB resistance in *Leishmania* parasites (10, 16, 17), this study assessed their involvement in the activity of APs. For this purpose, the AP susceptibility of nonresistant strains in combination with reference ABC transport inhibitors was evaluated; probenecid is known to reverse the effects of MRP pumps (67, 68), while verapamil and cyclosporine inhibit efflux mediated by both the MRP and MDR pumps (69–71). Treatment with Sb^III^ was included as a positive control (21, 72–75) and confirmed an increased susceptibility of extracellular and intracellular parasites in combination with verapamil and probenecid, although verapamil is considered not to efficiently inhibit the MDR pumps in *Leishmania* (76). Due to host cell toxicity, cyclosporine was included at a low concentration, which may explain the observed lack of an effect. The different response of L. donovani toward probenecid in combination with Sb^III^ could be explained by differences in pump expression between L. infantum and L. donovani (17, 77, 78). No significant effects could be noted after coincubation of the APs with the ABC transporter inhibitors in both extracellular and intracellular assays. The APs were also unable to inhibit the efflux of the fluorescent ABC efflux substrate 5(6)-carboxy-2′-7′-dichlorofluorescein.

To conclude, the DND*i* aminopyrazole lead series is promising when it comes to the likelihood of drug resistance development in the field, since repeated exposure of amastigotes to the compounds did not result in decreased susceptibility. Resistance selection of the promastigotes revealed that karyotypic changes may confer elevated levels of resistance, but these did not seem to be stable in the vertebrate and invertebrate hosts. Additionally, the AP lead compounds tested do not appear to be substrates of the MDR or MRP pumps of the parasite and macrophage host cell tested, reducing the risk of resistance development through this common pathway.

## MATERIALS AND METHODS

### Ethics statement.

The use of laboratory rodents was carried out in accordance with all mandatory guidelines (EU directives, including Revised Directive 2010/63/EU on the protection of animals used for scientific purposes, which came into force on 1 January 2013, and the Declaration of Helsinki [in its latest version]) and was approved by the Ethical Committee of the University of Antwerp, Antwerp, Belgium (UA-ECD 2011-77, revised 2015).

### Parasite cultures.

Promastigotes of two laboratory strains, MHOM/MA/67/ITMAP263 (Leishmania infantum) and MHOM/ET/67/L82 (L. donovani), were cultured at 25°C in HOMEM (Gibco, Life Technologies) supplemented with 10% inactivated bovine serum (iFBS). Both strains were also available as *ex vivo* amastigotes obtained from the spleens of heavily infected donor hamsters (38). Additionally, two L. infantum field strains, MHOM/FR/09/LEM4038 and MHOM/FR/96/LEM3323, available as promastigotes, were obtained from HIV-infected patients (79).

### Animals.

Female Swiss mice, BALB/c mice, and female golden hamsters were purchased from Janvier (Le Genest Saint Isle, France). Food for laboratory rodents (Carfil, Arendonk, Belgium) and drinking water were available *ad libitum*. Before the start of the *in vivo* experiments, the hamsters were randomly allocated to experimental units of 3 animals each.

### Test substances.

For the *in vitro* experiments, the aminopyrazoles (see Table S1 in the supplemental material) were formulated in 100% dimethyl sulfoxide (DMSO) at 20 mM (stock solution). The efflux pump inhibitors verapamil (an MDR and MRP inhibitor), cyclosporine (a broad-specificity efflux inhibitor), and probenecid (an MRP inhibitor) (Sigma-Aldrich, Diegem, Belgium) were formulated in 100% DMSO at 20 mM, except for probenecid, which was diluted in phosphate-buffered saline (PBS) after the addition of ethanol (2%) and NaOH at 50 mM. Stock solutions were further diluted in demineralized water. In all *in vitro* assays, the final in-test concentration of DMSO did not exceed 1%. For the *in vivo* experiments, DNDI-1044 was formulated in 1% (wt/vol) methylcellulose (4,000 centipoise) and 5% (vol/vol) Tween 80 in water; miltefosine (MIL) was formulated in water.

### Intracellular susceptibility assay.

To obtain primary peritoneal macrophages, Swiss mice were stimulated by intraperitoneal injection of 1 ml of a 2% starch suspension in PBS 48 h prior to cell collection. Animals were euthanized with a CO_2_ overdose, and upon removal of the skin, 10 ml of RPMI 1640 (Life Technologies) was injected into the peritoneal cavity to collect the macrophages, which were then seeded into 96-well plates at a final concentration of 30,000 cells/well in 100 μl of RPMI 1640 medium supplemented with 5% iFBS, 2% penicillin-streptomycin, and 1% l-glutamine (Life Technologies). After 24 h, the cells were infected with metacyclic promastigotes (infection ratio, 15:1), and 2-fold drug dilutions were added 24 h later. The test plates were incubated for 96 h at 37°C in 5% CO_2_, after which the cells were fixed with methanol and Giemsa stained for microscopic determination of the IC_50_ values, based on the reduction in the amastigote burden in the treated cells compared to that in the untreated control cells.

### Extracellular susceptibility assay.

Log-phase promastigotes (3 days old) were counted in a Kova counting chamber and diluted to a concentration of 10^6^ promastigotes/well in a 96-well plate. Dilutions of the test compounds were then added to the promastigote plates. The highest in-test concentrations and dilutions of all the compounds were as described above. Drug exposure covered a 72-h period without renewal of the culture medium. Parasite proliferation was assessed with the resazurin assay. Drug activity was measured as the percent reduction in the mean fluorescence compared to that for the nontreated control wells.

### *In vitro* intracellular resistance selection.

Intracellular amastigotes were repeatedly exposed to DNDI-1044, as previously described (39). In brief, primary peritoneal mouse macrophages were infected with metacyclic L. infantum promastigotes (MHOM/FR/09/LEM4038 and MHOM/MA/67/ITMAP263) in two duplicate 96-well plates and exposed to increasing drug concentrations. One plate was Giemsa stained to determine the drug susceptibility by microscopic evaluation, while the other plate was used for promastigote backtransformation (PBT). PBT was evaluated by releasing residual viable amastigotes and allowing their transformation into promastigotes in HOMEM at 25°C. Promastigotes were collected from the highest concentration and further expanded in routine culture. Infection and PBT cycles were repeated until resistance could be observed or for a maximum of five successive passages. Statistically significant differences between the different passages were analyzed using two-way analysis of variance (ANOVA), and the results were considered statistically significantly different if *P* was <0.05.

### *In vitro* extracellular resistance selection.

Log-phase promastigotes of the Leishmania infantum clinical isolate MHOM/FR/96/LEM3323 (3 days old) were counted and diluted to a concentration of 1 × 10^6^ promastigotes/ml containing 0.5× the IC_50_ of DNDI-1044 or DNDI-8012. Promastigotes were left to recover from drug exposure without renewal of the medium. Upon complete recovery, a next selection round was initiated by subculturing at twice the drug concentration of the previous selection round. Thus, promastigotes were gradually exposed to increasing drug concentrations until their growth was irreversibly inhibited. A sample from each selection cycle was cryopreserved in liquid nitrogen upon supplementation with 10% glycerol. Finally, intracellular and extracellular susceptibility assays were performed to assess the phenotypic acquisition of resistance as a result of the selection procedure. Statistically significant differences between the different passages were analyzed using two-way analysis of variance (ANOVA), and the results were considered statistically significantly different if *P* was <0.05.

### *In vivo* resistance selection.

The *in vivo* selection of drug resistance was performed as previously described (38). Briefly, L. infantum spleen-derived amastigotes (MHOM/MA/67/ITMAP263) were diluted to prepare an infection inoculum containing 2 × 10^7^ amastigotes in 100 μl PBS. All animals were treated orally for 5 days starting from 21 days postinfection (dpi), after which the animals were closely monitored for signs of relapse. Upon relapse, amastigotes were collected and used for infection of new naive hamsters, which received the same treatment regimen at 21 dpi. This procedure was repeated until evaluation of the *in vitro* IC_50_ values revealed a reduced drug susceptibility or for a maximum of 5 treatment/relapse cycles. A treatment regimen of 50 mg/kg of body weight twice a day (b.i.d.) was chosen, based on previously published results, to ascertain a parasite clearance of more than 95% in the main target organs (28). DNDI-1044 at 50 mg/kg b.i.d. was previously shown to result in an amastigote burden reduction of 97.1% and 98.6% 10 days after the end of treatment in liver and spleen, respectively. Statistically significant differences between the different passages were analyzed using two-way analysis of variance (ANOVA). Results were considered statistically significantly different if *P* was <0.05.

### Generation of clones.

The microdrop technique was used to generate clones of the resistant lines to reduce genetic variability in the population (80). A dilution of the culture was prepared in HOMEM in a 96-well plate, from which microdrops were taken. A total of 8 μl HOMEM was placed on the sidewall of a new well plate before placing the drop in the middle of the well. The drop was then microscopically evaluated for the presence of a single promastigote(s), and 100 μl of HOMEM and 100 μl of spent medium were added to the well. The plates were incubated at 25°C and observed weekly for promastigote growth under a microscope. Once growth was established, the promastigotes were transferred into culture flasks and subcultured twice weekly according to standard procedures.

### Intracellular multiplication.

Amastigote multiplication was compared between the resistant clones and the wild-type parent line for assessment of a possible loss or gain of infectivity accompanied by the resistant phenotype. Peritoneal macrophages were collected and infected with metacyclic promastigotes at an infection ratio of 2:1 as described previously (43). Parasites were preconditioned in acidic HOMEM (pH 5.4) 24 h prior to infection to ascertain that all promastigotes were at the same metacyclic stage (81). The infected macrophages were washed twice with RPMI 1640 after 24 h of infection to exclude the possibility of reinfection by noninternalized promastigotes. The medium on the cells was replaced every 4 days to ensure macrophage viability. Amastigote multiplication was evaluated every day for a period of 7 days postinfection by microscopy evaluation after Giemsa staining. The infection index was calculated by dividing the total number of counted amastigotes by the total number of counted macrophages. The amastigote multiplication ratio was then calculated by dividing the infection index of every time point by the infection ratio at 24 hpi, thereby correcting for baseline infectivity differences.

### Stability of the resistant phenotype after sandfly passage.

The stability of the resistant phenotype was assessed for the most resistant clones compared to that for the wild-type parent line after passage in the vector. Two- to 5-day-old female Lutzomyia longipalpis sandflies were fed through a chick skin membrane on a mixture containing heparin-treated mouse blood and 5 × 10^6^ logarithmic-phase promastigotes per ml. Blood-fed sandflies were separated 24 h later and maintained at 26°C with 30% (vol/vol) sugar solution. Seven days after feeding, the flies were anesthetized with CO_2_, and their midguts were dissected and crushed after addition of 50 μl of PBS. The released parasites were then incubated in acidic HOMEM (pH 5.4) supplemented with 20% iFBS, 1% gentamicin, 2% penicillin-streptomycin, and 2% spent medium. After 24 to 48 h of incubation, parasites were subcultured and susceptibility testing was performed in both promastigotes and intracellular amastigotes.

### Stability of the resistant phenotype after mouse passage.

The stability of the resistant phenotype was also assessed after mouse passage. Infection was performed by injecting 1 × 10^8^ metacyclic promastigotes into the tail vein of 8-week-old BALB/c mice. After 3 weeks of infection, the mice were euthanized and PBT was performed from the liver, spleen, and bone marrow. This entailed incubating a small piece of organ (spleen, liver, bone marrow from the femur) in acidic HOMEM (pH 5.4) supplemented with 20% iFBS, 1% gentamicin, 2% penicillin-streptomycin, and 2% spent medium. After 5 days of incubation, promastigotes were subcultured and susceptibility testing was subsequently performed.

### Comparative whole-genome sequencing.

Genomic DNA was prepared from the L. infantum resistant clones (1044Cl_1_ and 8012Cl_2_) and the wild-type (WT) parent line. DNA was quantified fluorometrically, and 2 μg of material was used for library preparation and sequencing on an Illumina NovaSeq 6000 sequencer with a NovaSeq 6000 S2 reagent kit (200 cycles). The reads obtained were aligned against the L. infantum JPCM5 reference genome (Tritryp_v9) using BWA-MEM software. Single nucleotide polymorphisms (SNPs) and indels were identified using GATK software. SNPs and indels that were present in the WT line but that were not located in coding sequences or that did not generate an amino acid alteration in the resistant lines (synonymous mutations) were not considered. To determine the influence of the mutation, the allele frequency (homozygous/heterozygous), the impact of the mutation (as a property of the altered amino acid and indel), and the presence of the mutated gene in both resistant lines were taken into account. Genes with identical mutations in both resistant lines and genes harboring several mutations (≥3) were considered less important, as they likely reflect natural polymorphisms. Additionally, the copy number variations (CNVs) in each chromosome were determined by calculating the ratio of the number of normalized reads from the resistant line compared to the number from the susceptible parent line.

### Intracellular efflux susceptibility assay.

Macrophages were infected with spleen-derived L. infantum (MHOM/MA/67/ITMAP263) or L. donovani (MHOM/ET/67/L82) amastigotes at an infection ratio of 5:1. After 2 h, the medium was replaced with 220 μl of fresh RPMI 1640 medium containing the aminopyrazole and the ABC transporter inhibitor. The latter was added at a concentration below the IC_50_, as determined in the standard intracellular amastigote susceptibility assay (Table S2). The DND*i* compounds were diluted 4-fold, with the highest in-test concentration being 10 μM. Drug activity was evaluated as described above in the intracellular susceptibility assay, and IC_50_ values were compared for conditions with and without an ABC transporter inhibitor.

### Extracellular efflux susceptibility assay.

Log-phase promastigotes (3 days old) of L. infantum and L. donovani were diluted to a concentration of 10^6^ promastigotes/well in a 96-well plate. Dilutions of the reference compounds and aminopyrazoles were then added either with or without the ABC transporter inhibitor. The IC_50_ of the inhibitor was first determined by a standard promastigote susceptibility assay (Table S2). Drug activity was determined as described above for the extracellular susceptibility assay, and IC_50_ values were compared for conditions with and without an ABC transporter inhibitor.

### Flow cytometric analysis of drug efflux.

The accumulation of 5(6)-carboxy-2′-7′-dichlorofluorescein (CDCF; Sigma-Aldrich) was evaluated upon incubation of macrophages (2 × 10^5^ cells/measurement) with 160 μM CDCF in RPMI 1640 without phenol red for 1 h at 4°C. When experiments were performed with *Leishmania*-infected macrophages, the cells were first seeded in a T75 culture flask in RPMI 1640 medium and infected with *ex vivo* amastigotes for 24 h. After incubation, the cells were washed twice with cold medium and an aliquot was taken to quantify CDCF uptake by flow cytometry in the fluorescein isothiocyanate channel (FACSCalibur flow cytometer; Becton, Dickinson). Dye retention in macrophages was evaluated after 2 h of incubation at 37°C in the absence (negative control) or presence (positive control) of probenecid at 4 mM alongside the DND*i* compounds and Sb^III^ at 10 μM. To assess cell viability, 7-amino actinomycin D (7-AAD; Biosciences) was added. Data analysis and determination of the mean fluorescence intensity were carried out with Flow Jo X software.

## Supplementary Material

Supplemental file 1
