# Supplementary material for: Antileishmanial Aminopyrazoles: Studies into Mechanisms and Stability of Experimental Drug Resistance
Source: Antimicrob Agents Chemother. 2020 Aug 20;64(9):e00152-20. doi: 10.1128/AAC.00152-20 (PMC7449183; doi:10.1128/AAC.00152-20)
Supplement: Supplemental file 1 [file AAC.00152-20-s0001.pdf]

<u>**Supplementary data**</u>

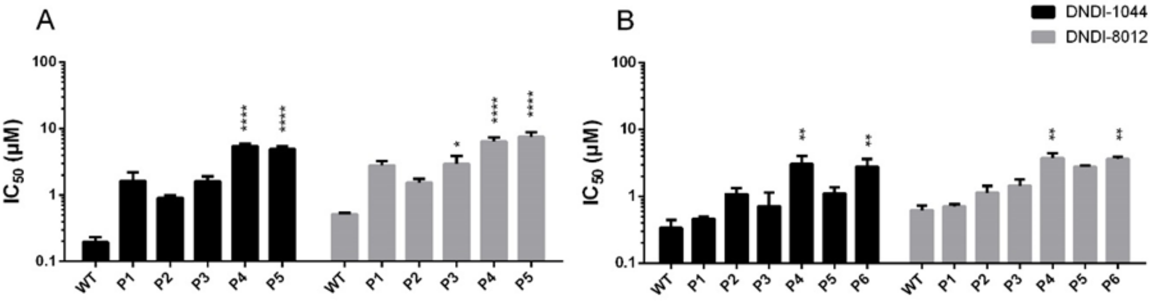

Figure S1: Comparison of the *in vitro susceptibility* in the <u>extracellular promastigote assay</u> of the wild type parent (WT) with selected lines LEM3323Cl$_4$/1044 (A) and LEM3323Cl$_4$/8012 (B) at each passage following exposure to the APs DNDI-1044 and DNDI-8012. Results are expressed as the mean IC_{50} (µM) ± SEM and are based on two independent replicates run in duplicate. (*: $p < 0.05$, **: $p < 0.01$, ****: $p < 0.0001$).

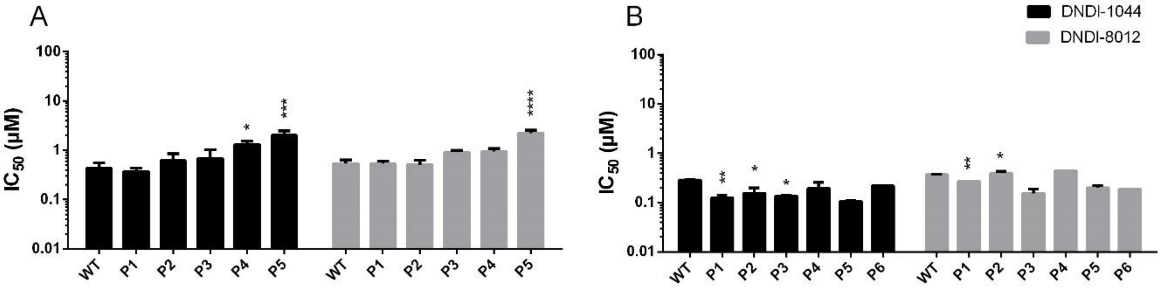

Figure S2: Comparison of the *in vitro* susceptibility in the <u>intracellular amastigote assay</u> of the wild type (WT) parent with selected lines LEM3323Cl$_4$/1044 (A) and LEM3323Cl$_4$/8012 (B) at each passage following exposure to the APs DNDI-1044 and DNDI-8012. Results are expressed as the mean IC_{50} (µM) ± SEM and are based on two independent replicates run in duplicate. (*: $p < 0.05$, **: $p < 0.01$, ***: $p < 0.001$, ****: $p < 0.0001$).

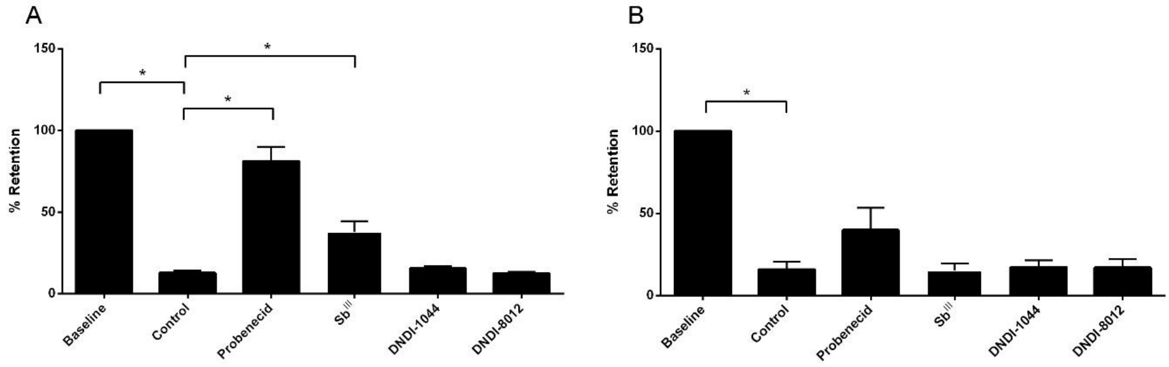

Figure S3: Percentage retention of 5(6)-carboxy-2'-7'dichlorofluorescein (CDCF) in uninfected (A) and infected (B) macrophages after 2h of efflux with probenecid or the aminopyrazoles. Results are based on two independent repeats run in duplicate and are expressed as the mean of the IC_{50} ± the standard mean of error (SEM) (*p <0.05).

Table S1. Chemical structure of the aminopyrazole series.

DNDI-1044	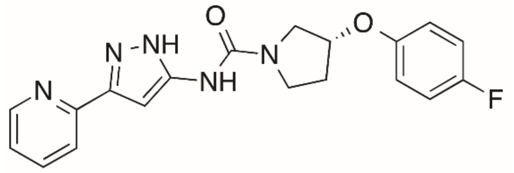
DNDI-8012	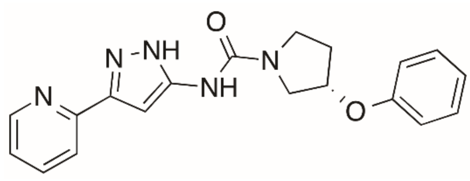
DNDI-5561	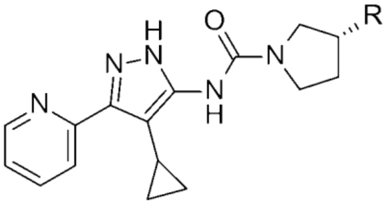

Table S2: Mean macrophage infection rate and infection ratio of both the resistant strains compared to the wild type line at 24hpi. Results are expressed as the mean percentage infected macrophages or infection ratio (#amastigotes/#macrophages) ± standard error of mean (SEM) and are based on one experiment run in sextuplet.

24hpi	$LEM3323Cl_4$	$LEM3323Cl_4/1044Cl_1$	$LEM3323Cl_4/8012Cl_2$
**% Infected macrophages**	87 ± 3	86 ± 2	87 ± 5
**Infection ratio**	6.2 ± 0.8	7.7 ± 0.6	6.1 ± 0.8

Table S3: *In vitro* activity of the efflux pump inhibitors on intracellular amastigotes and extracellular promastigotes of the *L. infantum* and *L. donovani* laboratory strains. Results are expressed as the mean IC_{50} (µM) ± standard error of mean (SEM) and are based on two independent replicates run in duplicate (*n*=4).

		Verapamil	Cyclosporine A	Probenecid
**Intracellular amastigote assay**	*L. infantum*	10 µM	2 µM	> 4.5 mM
	L. donovani	10 µM	4 µM	> 4.5 mM
	Cytotoxicity	64 µM	10 µM	> 4.5 mM
**Extracellular promastigote assay**	*L. infantum*	100 µM	14 µM	875 µM
	L. donovani	40 µM	15 µM	875 µM

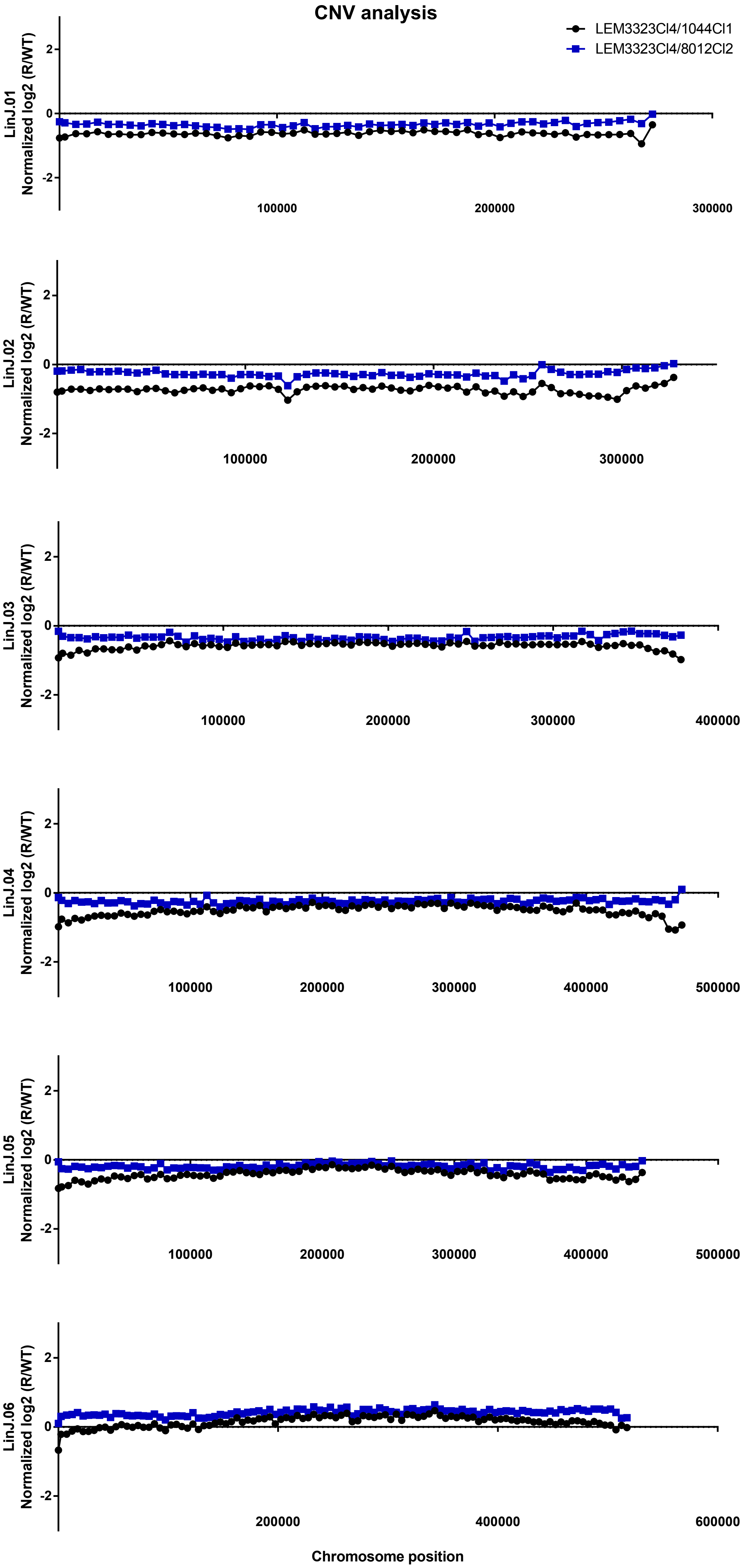

CNV analysis
LEM3323Cl4/1044Cl1
LEM3323Cl4/8012Cl2
LinJ.01
LinJ.02
LinJ.03
LinJ.04
LinJ.05
LinJ.06
Normalized log2 (R/WT)
2
0
-2
100000
200000
300000
400000
500000
600000
Chromosome position

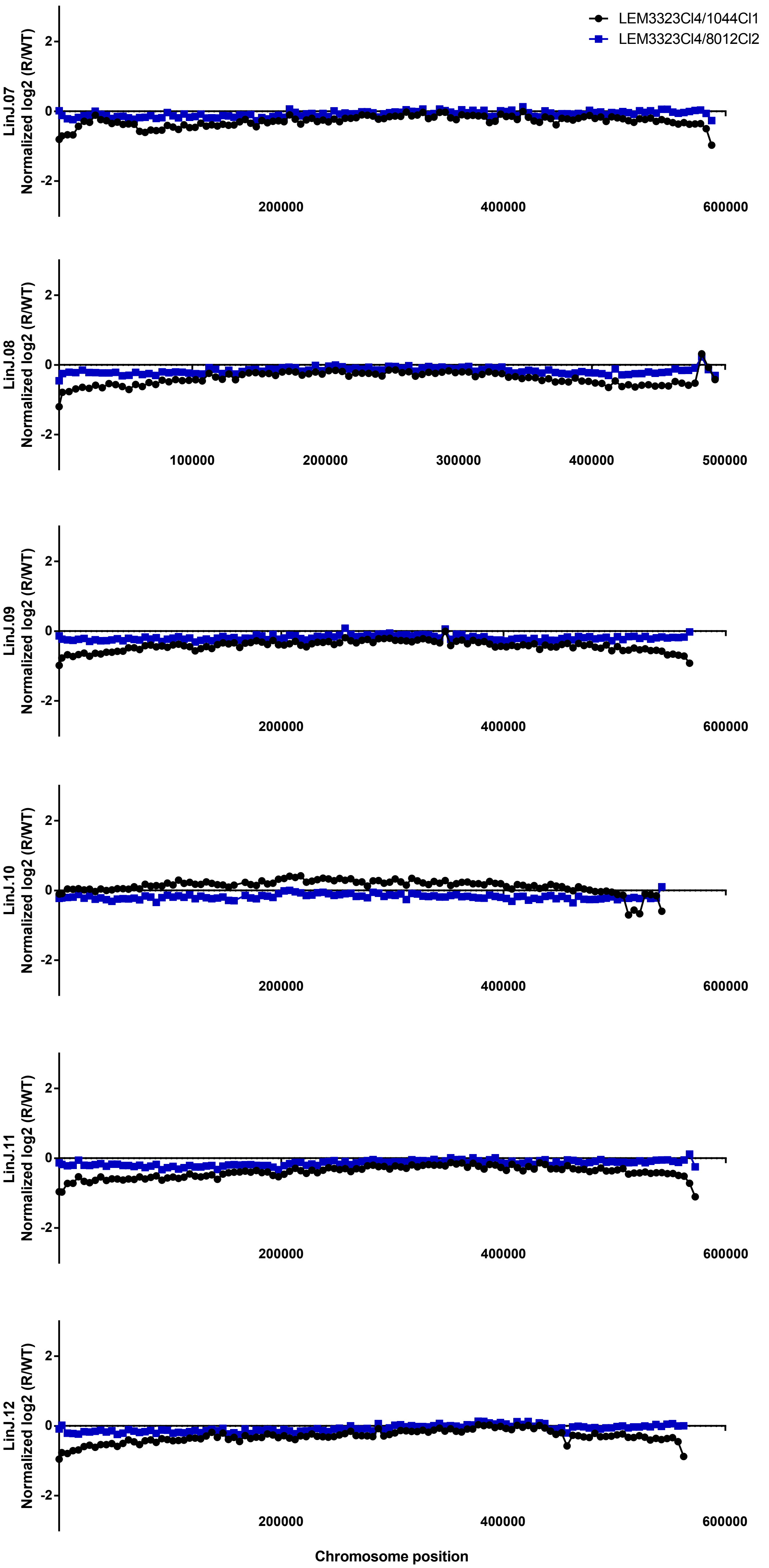
LEM3323Cl4/1044Cl1
LEM3323Cl4/8012Cl2
LinJ.07
LinJ.08
LinJ.09
LinJ.10
LinJ.11
LinJ.12
Normalized log2 (R/WT)
2
0
-2
100000
200000
300000
400000
500000
600000
Chromosome position

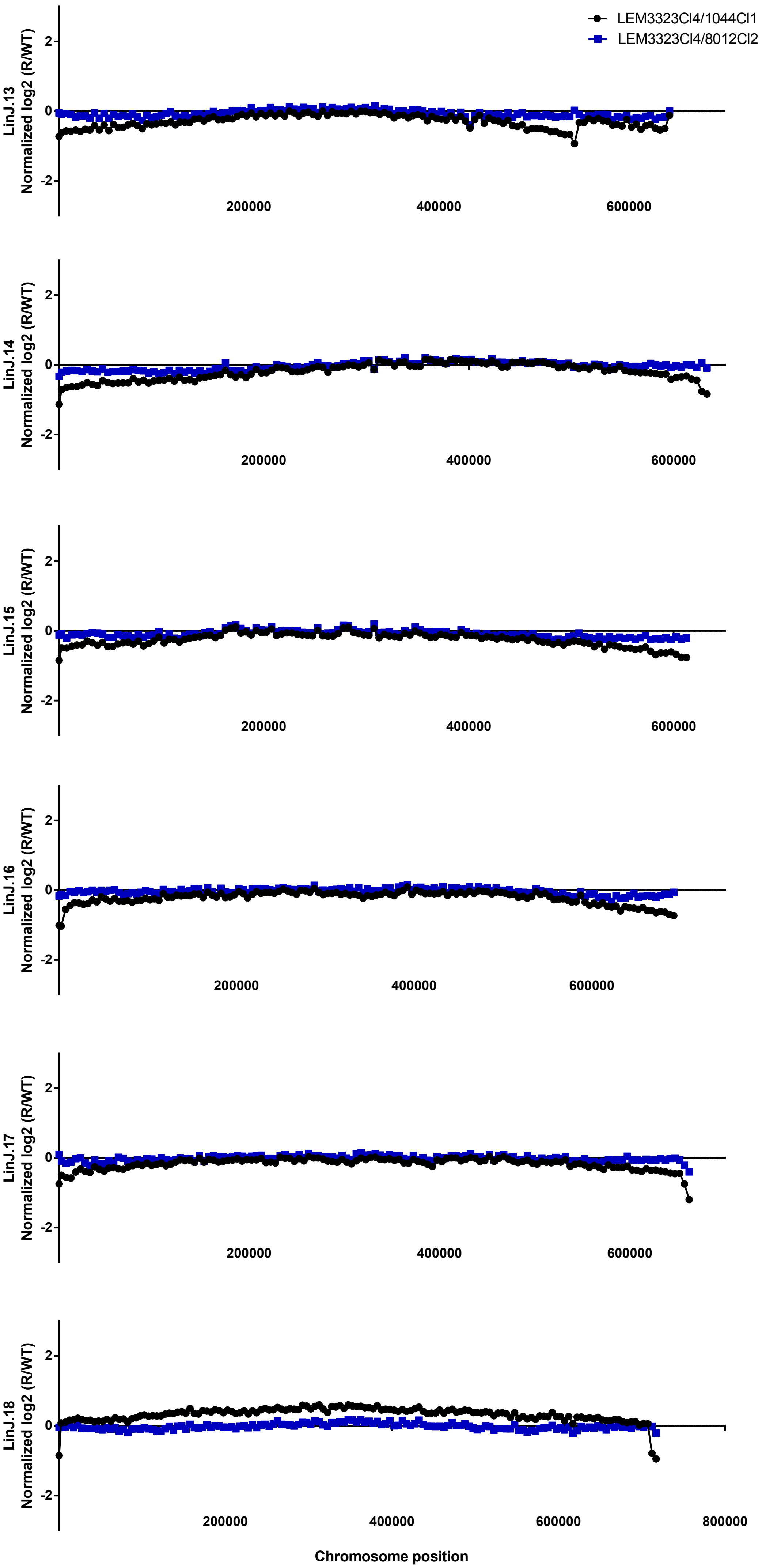

LEM3323Cl4/1044Cl1
LEM3323Cl4/8012Cl2
LinJ.13
LinJ.14
LinJ.15
LinJ.16
LinJ.17
LinJ.18
Normalized log2 (R/WT)
2
0
-2
200000
400000
600000
800000
Chromosome position

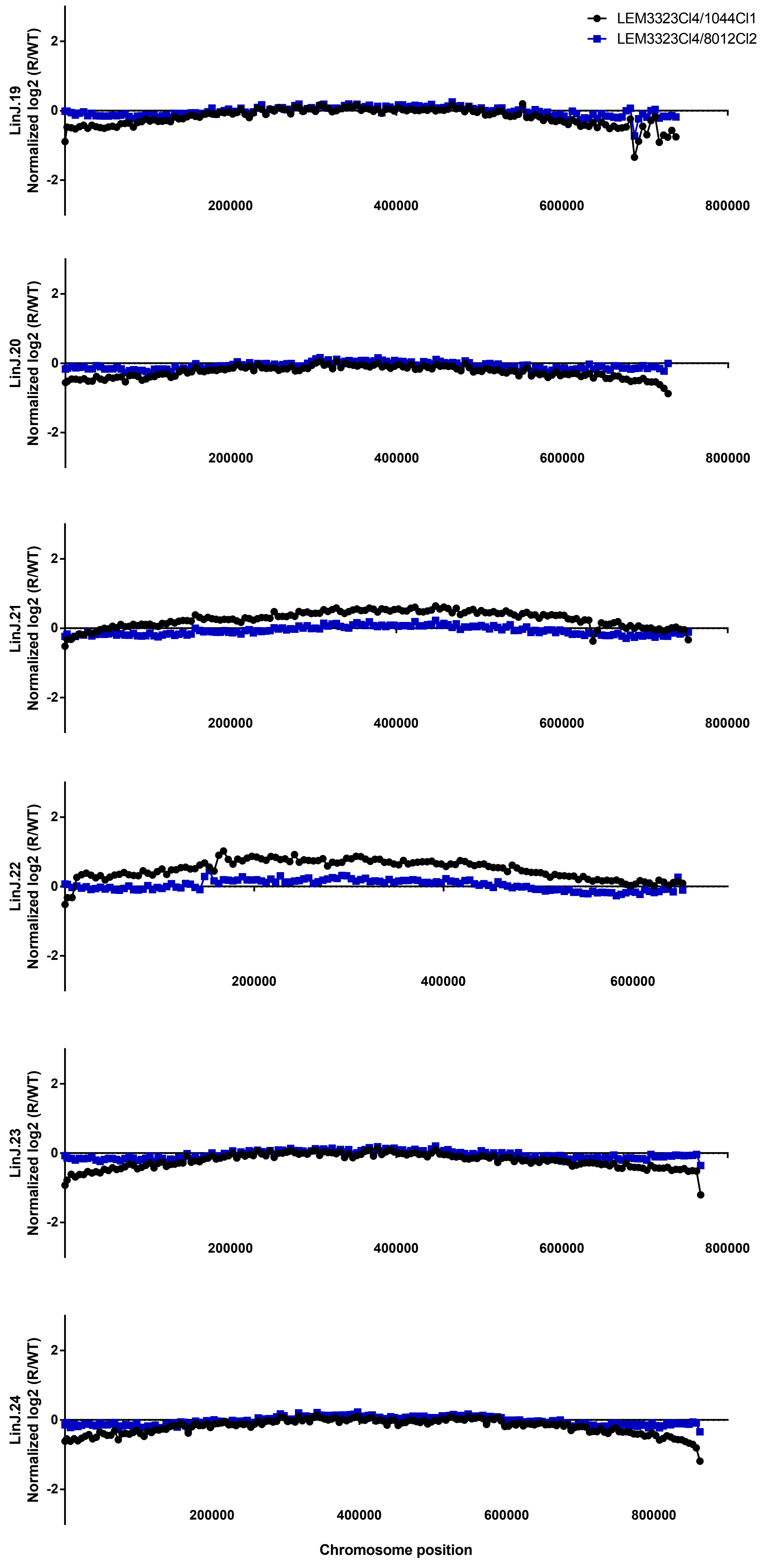

LEM3323Cl4/1044Cl1
LEM3323Cl4/8012Cl2
LinJ.19
LinJ.20
LinJ.21
LinJ.22
LinJ.23
LinJ.24
Normalized log2 (R/WT)
2
0
-2
200000
400000
600000
800000
Chromosome position

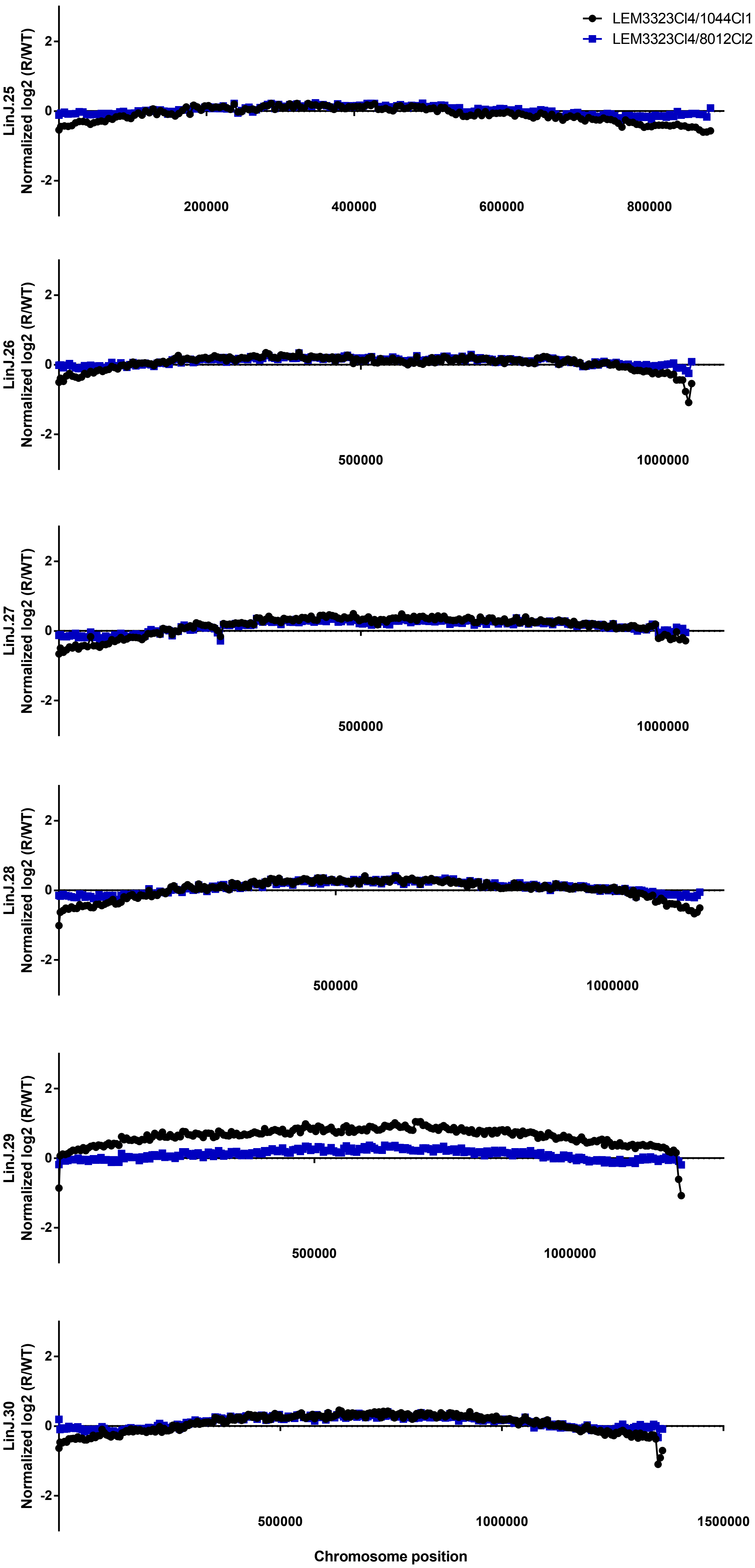
LEM3323Cl4/1044Cl1
LEM3323Cl4/8012Cl2
LinJ.25
Normalized log2 (R/WT)
2
0
-2
200000
400000
600000
800000
LinJ.26
Normalized log2 (R/WT)
2
0
-2
500000
1000000
LinJ.27
Normalized log2 (R/WT)
2
0
-2
500000
1000000
LinJ.28
Normalized log2 (R/WT)
2
0
-2
500000
1000000
LinJ.29
Normalized log2 (R/WT)
2
0
-2
500000
1000000
LinJ.30
Normalized log2 (R/WT)
2
0
-2
500000
1000000
1500000
Chromosome position

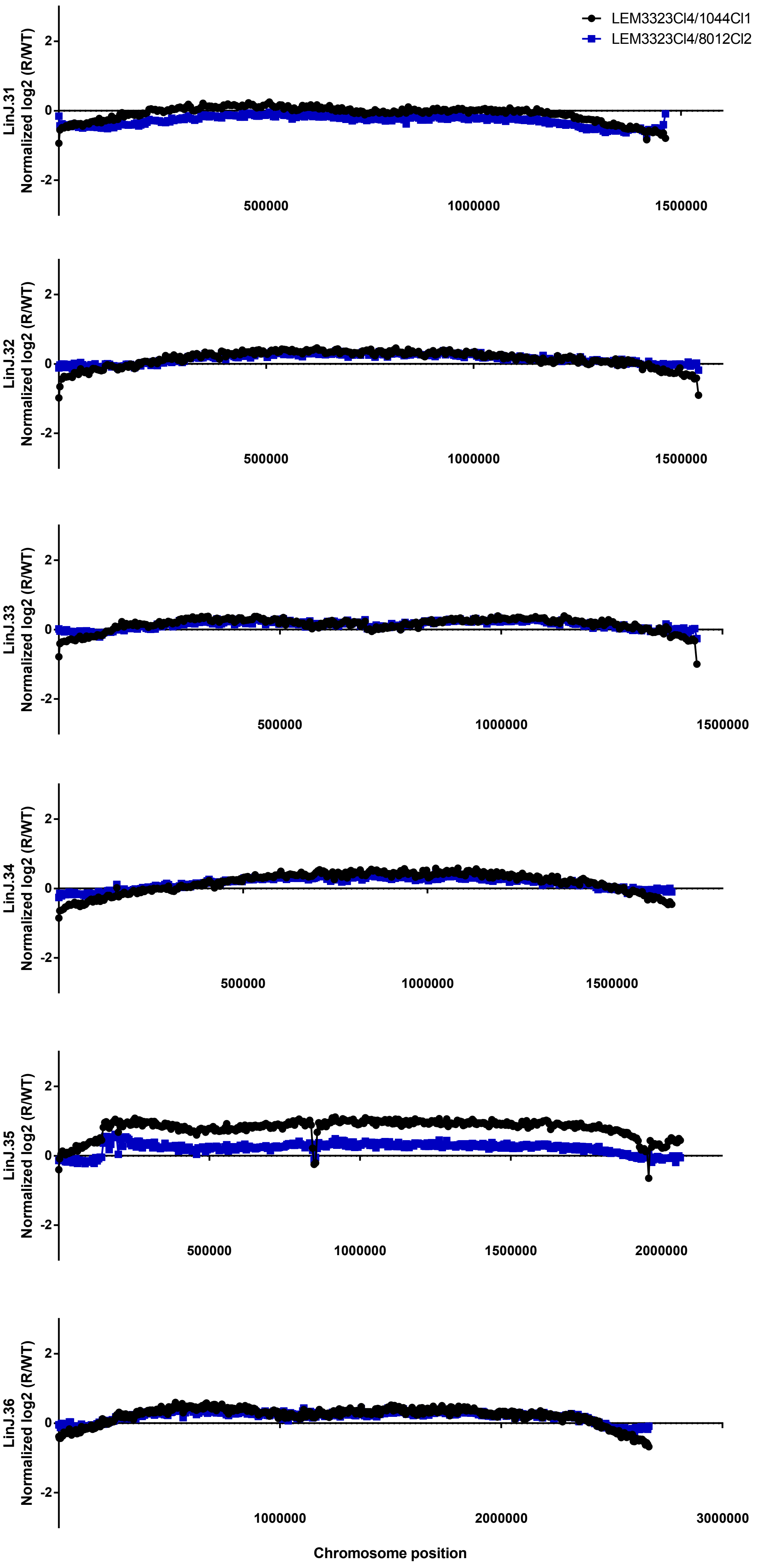

LEM3323Cl4/1044Cl1
LEM3323Cl4/8012Cl2
LinJ.31
LinJ.32
LinJ.33
LinJ.34
LinJ.35
LinJ.36
Normalized log2 (R/WT)
2
0
-2
500000
1000000
1500000
2000000
3000000
Chromosome position
